# In this month’s *Bulletin*

**DOI:** 10.2471/BLT.15.000515

**Published:** 2015-05-01

In the editorial section, Flavia Bustreo and Robin Gorna (286) call for papers that link millennium to sustainable development goals on women’s, children’s and adolescents’ health. Lisa G Johnston et al. (287) discuss ways to obtain information on HIV status; a topic debated in a round table led by Rachel Baggaley et al. (353–358).

In the news section, Gary Humphreys (290–291) reports on a technology transfer collaboration between Argentina and the Netherlands. Fiona Fleck interviews Adetokunbo Lucas (292–293) about the five decades he has spent working in public health.

## New Zealand

## Legislating for better food

Stefanie Vandevijvere et al. (294–302) study implementation of healthy food policies.

## South Africa

## Correcting the number of deaths

Richard Matzopoulos et al. (303–313) collect data on deaths caused by injuries.

## Comoros, Eritrea, Ethiopia, Kenya, Lesotho, Malawi, Mozambique, Namibia, Rwanda, Swaziland, Uganda, Zambia & Zimbabwe

## Measuring measles vaccinations

Reinhard Kaiser et al. (314–319) review vaccination coverage survey reports

## Gambia

## Getting to yes

Muhammed Olanrewaju Afolabi et al. (320–328) test a multimedia consent tool for research participants.

## Japan

## Correcting global estimates with national data

Michael Roerecke et al. (329–338) recalculate national estimates of alcohol-related oesophageal cancer.

## Zimbabwe

## Preventing maternal and neonatal deaths

Joanna F Crofts et al. (347–352) train hospital staff to confront obstetric emergencies.

## Global

## Building demand for vaccines

Mira Johri et al. (339–346) review strategies designed to increase demand for childhood vaccinations.

## Reducing asbestos exposure

Oladele A Ogunseitan (359–360) documents missed opportunities to prevent asbestos-related diseases.

